# Association between Daily Hydrogen Sulfide Exposure and Incidence of Emergency Hospital Visits: A Population-Based Study

**DOI:** 10.1371/journal.pone.0154946

**Published:** 2016-05-24

**Authors:** Ragnhildur Gudrun Finnbjornsdottir, Hanne Krage Carlsen, Throstur Thorsteinsson, Anna Oudin, Sigrun Helga Lund, Thorarinn Gislason, Vilhjalmur Rafnsson

**Affiliations:** 1 Centre of Public Health Sciences, University of Iceland, Stapi, v/Hringbraut, 101 Reykjavik, Iceland; 2 Occupational and Environmental Medicine, Department of Public Health and Clinical Medicine, Umeå University Hospital, 901 85 Umeå, Sweden; 3 Environment and Natural Resources, University of Iceland, Sturlugötu 7, 101 Reykjavik, Iceland; 4 Faculty of Medicine, University of Iceland, Vatnsmýrarvegur 16 v/Landspítala, 101 Reykjavik, Iceland; 5 Department of Respiratory Medicine and Sleep, Landspitali University Hospital, Fossvogi, 108 Reykjavik, Iceland; 6 Department of Preventive Medicine, University of Iceland, Stapi, v/Hringbraut, 101 Reykjavik, Iceland; National Institute of Agronomic Research, FRANCE

## Abstract

**Background:**

The adverse health effects of high concentrations of hydrogen sulfide (H_2_S) exposure are well known, though the possible effects of low concentrations have not been thoroughly studied. The aim was to study short-term associations between modelled ambient low-level concentrations of intermittent hydrogen sulfide (H_2_S) and emergency hospital visits with heart diseases (HD), respiratory diseases, and stroke as primary diagnosis.

**Methods:**

The study is population-based, using data from patient-, and population-registers from the only acute care institution in the Reykjavik capital area, between 1 January, 2007 and 30 June, 2014. The study population was individuals (≥18yr) living in the Reykjavik capital area. The H_2_S emission originates from a geothermal power plant in the vicinity. A model was used to estimate H_2_S exposure in different sections of the area. A generalized linear model assuming Poisson distribution was used to investigate the association between emergency hospital visits and H_2_S exposure. Distributed lag models were adjusted for seasonality, gender, age, traffic zones, and other relevant factors. Lag days from 0 to 4 were considered.

**Results:**

The total number of emergency hospital visits was 32961 with a mean age of 70 years. In fully adjusted un-stratified models, H_2_S concentrations exceeding 7.00μg/m^3^ were associated with increases in emergency hospital visits with HD as primary diagnosis at lag 0 risk ratio (RR): 1.067; 95% confidence interval (CI): 1.024–1.111, lag 2 RR: 1.049; 95%CI: 1.005–1.095, and lag 4 RR: 1.046; 95%CI: 1.004–1.089. Among males an association was found between H_2_S concentrations exceeding 7.00μg/m^3^, and HD at lag 0 RR: 1.087; 95%CI: 1.032–1.146 and lag 4 RR: 1080; 95%CI: 1.025–1.138; and among those 73 years and older at lag 0 RR: 1.075; 95%CI: 1.014–1.140 and lag 3 RR: 1.072; 95%CI: 1.009–1.139. No associations were found with other diseases.

**Conclusions:**

The study showed an association between emergency hospital visits with HD as primary diagnosis and same day H_2_S concentrations exceeding 7.00μg/m^3^, more pronounced among males and those 73 years and older than among females and younger individuals.

## Introduction

The adverse health effects of high concentrations of hydrogen sulfide (H_2_S) exposure are many and relatively well known, as has been reviewed in a report by the World Health Organization [1], but the mechanisms of H_2_S toxicity remain debated. Some studies indicate that H_2_S inhibit oxygen consumption by mitochondrial oxidase [2], and others suggest that H_2_S may affect cysteine residues of most proteins [3]. The first noticeable effect of H_2_S is the odour similar to rotten eggs; the odour threshold varies, often considered 7–11 μg/m^3^ [1,4,5]. With increasing H_2_S concentrations other effects appear, for example, eye irritation and neurological symptoms such as headache, nausea, loss of olfactory sense (at 140 mg/m^3^) [1]. Pulmonary oedema, respiratory arrest, and death may follow a few breaths at 700 mg/m^3^ [1].

Studies on low-level H_2_S exposures have been accumulating through observations of occupational cohorts and populations residing near industries and geothermal fields emitting H_2_S and other pollutants [6–13]. These studies have dealt with different outcomes; some have reported association with noticing odour, odour nuisance and decreased daily activity [6,7], increase in respiratory symptoms and anti-asthma drug dispensing [7–9], while others have reported negative associations between long-term H_2_S exposure and self-reported asthma and asthma symptoms [10]. Still other studies have reported on neurological symptoms and headaches [8,11,12] while the results on the effect of H_2_S exposure on cognitive function remains inconclusive [12,13]. Respiratory mortality and total mortality, as well as lung cancer, have been associated with low-level H_2_S exposures [14–16]. Reduced lung function has been reported in two studies [11,17], but was not found in one study [18]. Finally, visits to health care centres and hospitals have been used to study H_2_S-exposed catchment populations with emphasis on respiratory diseases and cardiovascular diseases where five studies report positive associations [19–22], while a recent study that attempted to evaluate long-term exposure found no association [23].

The comprehensive hospital and population registries operated in Iceland offer a unique opportunity for population-based studies on low-level H_2_S exposed inhabitants in the Reykjavik capital area. Since 2006, two geothermal power plants have been located some 30 km east of the city and the characteristic odour of H_2_S is occasionally noticed in Reykjavik. The H_2_S concentrations have been measured in the capital area with a total population of approximately 196,000 individuals [24].

The aim was to investigate short-term associations between modelled ambient low-level intermittent H_2_S concentrations and daily hospital admissions and emergency department (ED) visits to Landspitali University Hospital (LUH) with heart disease, respiratory disease and stroke as primary diagnoses among individuals living in the Reykjavik capital area.

## Materials and Methods

### Study population

Reykjavik is the world’s northernmost capital of a sovereign state and is located in the south-west of Iceland on the southern shore of the Faxafloi bay.

The study period was 1 January 2007 to 30 June 2014. The National Roster, part of the National Registry, kept by Statistics Iceland, was the source of information on the population base (number, age, and gender) which consisted of all individuals, 18 years and older in the Reykjavik capital area. The population was geocoded into sections A to E (see Exposure assessment subchapter) and the total number of individuals in each section (A-E) were calculated within age groups (18-59, 60-72, 73-80, and 81 and older) and gender. The Reykjavik capital area consists of seven municipalities (Alftanes, Gardabaer, Hafnarfjordur, Kopavogur, Mosfellsbaer, Reykjavik, and Seltjarnarnes), defined by community codes and 21 postal codes: 101, 103-105, 107-113, 170, 200, 201, 203, 210, 220, 221, 270, 271, and 276, according to the National Roster 2010 [24].

### Outcome measures

The primary source of data is the records on emergency hospital admissions and ED visits to the only acute care hospital and ED in the Reykjavik capital area at LUH, obtained from the Register of Hospital-treated Patients in Iceland for the study period. Patient data were anonymized and de-identified by LUH specialist prior to data handling. The hospital is operated by the government, and health-care services are financed by taxes. Residents of Iceland are covered by the national health insurance schemes, which pay the bulk of the patients’ costs; however, patients pay a certain fee for ambulatory visits. Admission to the hospital is free of charge. The register of Hospital-treated Patients is practically complete, and contains routinely collected data on every patient admission to the hospital and visit to the ED of those 18 years of age or older. Information registered includes the unique registration number of every admission and visit, personal identification numbers according to the National Registry, address, postal code, birth date, gender, admission date, discharge date and discharge diagnoses as diagnosed by the attending physician using the International Classification of Diseases 10th version (ICD-10).

The outcome measure was acute hospital admission, or visit to the ED, reported with one of the following classes of disease: heart disease (HD) (ICD-10 codes: I20-I27: ischaemic heart diseases, I46: cardiac arrest, I48: cardiac arrhythmias, and I50: heart failure), respiratory disease (ICD-10 codes: J20-J22: acute lower respiratory infections, J40-J46: chronic lower respiratory diseases, and J96: respiratory failure) and stroke (ICD-10 codes: I61-I69: cerebrovascular diseases other than I60: subarachnoid haemorrhage and G45: transient cerebral ischaemic attacks and related syndromes and G46: vascular syndromes of brain in cerebrovascular diseases), all as primary diagnosis. The daily number of acute hospital admissions and ED visits were combined and are henceforth referred to as “emergency hospital visits”. Encrypted personal identification numbers were used to find individuals with readmissions or revisits within 10 days and with same ICD-10 primary diagnosis; these revisits were excluded, and only the previous admission or ED visit was counted as a visit.

**Fig 1:**
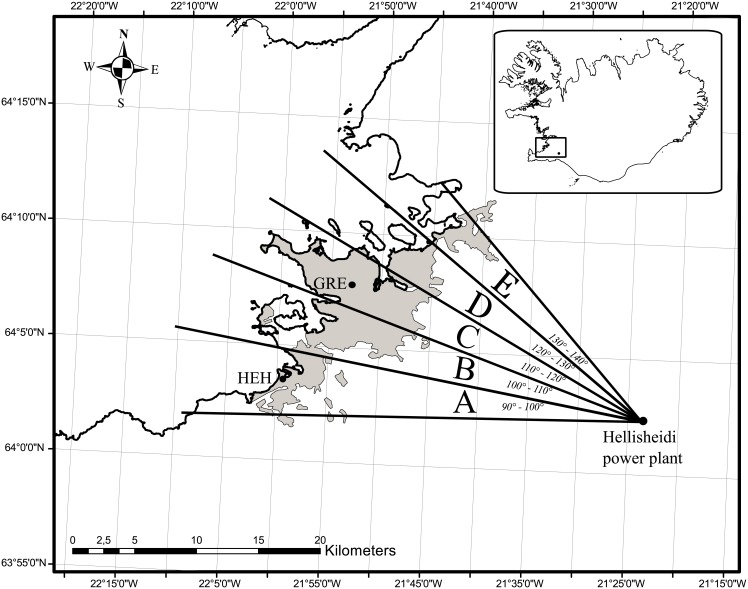
Five modelled sections (A to E) of the Reykjavik capital area (the shadowed area), and the point source of H_2_S emissions, the Hellisheidi power plant. Small inserted map shows Iceland and the capital’s location.

The population was divided into the geographical sections A to E by geocoding the addresses, and the National Roster was used to count the number of people at risk in the sections according to gender and age groups. Patients with an emergency hospital visit were tracked through home address and geocoded to the exact section A to E, date, and thus assigned H_2_S exposure. The parts of the population and patients located outside the borders of section A, and E, were counted with the adjoining sections; see next subchapter and Fig 1.

### Exposure assessment

For the study period 1 January, 2007 to 30 June, 2014, ambient air concentrations and meteorological data were obtained from the Environment Agency of Iceland (EAI), which operates a measurement station located near one of Reykjavik’s busiest road intersections (Grensasvegur station, GRE) [25]. The data contained hourly concentration values of nitrogen dioxide (NO_2_), ozone (O_3_), particulate matter ≤ 10 μm in aerodynamic diameter (PM_10_), sulfur dioxide (SO_2_) and H_2_S measured as micrograms per cubic metre of air (μg/m^3^) as well as hourly values of temperature (°C), relative humidity (RH, %), wind speed (m/s), and wind direction. The devices used to measure pollutant concentrations and calibration frequency have previously been reported [16].

Distance from main roads (>10.000 cars per day) in the Reykjavik capital area was found for each individual’s residential street and divided into categories of traffic exposure zones (0-50 m, 51-200 m, 201-500 m, 501-1000 m, and ≥1000 m) and used as a surrogate for traffic-related exposure. Measured air pollution concentrations from the GRE station, were not included in the final analysis as the exposure zones gave a better fit in the final analysis.

The main source of ambient H_2_S is from a geothermal power plant located 26 km east of the city centre (Fig 1) [26,27]. Hellisheidi power plant started operation in September 2006. Average H_2_S emissions over the study period were 10,532.5 tons annually, fluctuating between 6,902 tons/year in 2007 and 13,340 tons/year in 2010 [28]. Residential distance from the Hellisheidi power plant was adjusted for in the final analysis, by classifying the distance into quartiles (≤22 430 m, 22 431-25 360 m, 25 361-27 330 m, and ≥27 331 m).

To estimate H_2_S exposure through 2007 to July 2014 in different sections of the Reykjavik capital area, a simple model was applied whereas the modelled concentration only depends on wind speed, the angle between wind direction and modelled location, and incoming solar radiation. The width of the plume was determined from measurements and calculations using the well known Gaussian plume, Pasquill-Gifford model [29,30], at 25 km from the source under stable conditions [31]. The model predicted H_2_S concentrations that were compared to measured concentrations at measurement stations operated by EAI, in section A (Hvaleyrarholt station, HEH) and in section C (GRE) (Fig 1). Emissions from the Nesjavellir power plant were not included in the model, as the power plant is behind a mountain [31], which limits the distribution of H_2_S westward in the direction of the Reykjavik capital area [26,27,32], and this was confirmed by H_2_S measurement at GRE before the start of the Hellisheidi geothermal power plant in 2006 [16]. The model covers a 50° section from Hellisheidi power plant to the west, which includes the Reykjavik capital area. The concentration was calculated in five 10° sections, defined as A to E (Fig 1). For each section, the average 24-hour H_2_S concentration was calculated. The location of Hellisheidi power plant is some 260m above sea level and there is a moderate, practically continuous downward slope [31] westward from the plant to the Reykjavik capital area (GRE). Detailed description of the H_2_S modelling can be found in S1 Model Calculations. Model prediction and accuracy was considered sufficient with a Spearman’s correlation coefficient of 0.55 for daily averages of H_2_S concentrations (Figures D and E in S1 Model Calculations).

Different exposure levels of H_2_S were calculated by different percentiles 50% (2.46 μg/m^3^), 60% (3.16 μg/m^3^), 70% (4.14 μg/m^3^), 80% (5.74 μg/m^3^), 85% (7.00 μg/m^3^), 90% (8.80 μg/m^3^) and 95% (11.68 μg/m^3^), and trend analyses were conducted through the percentile levels.

### Statistical analysis

**Fig 2:**
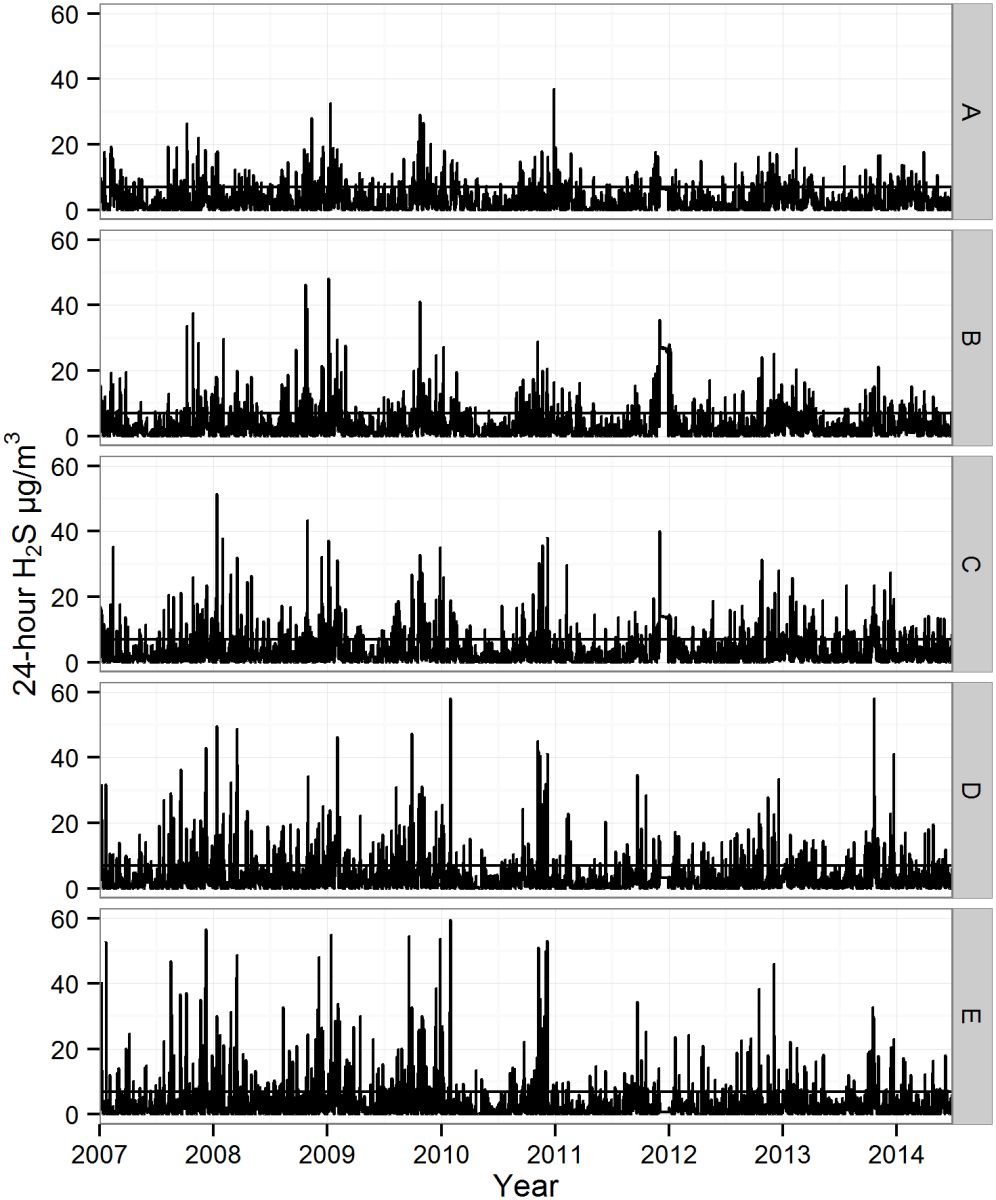
Daily 24-hour concentrations of H_2_S in μg/m^3^ within modelled sections A to E of the Reykjavik capital area over the study period 1 January, 2007–30 June, 2014. Horizontal line indicates the 85 percentile limit of 7.00 μg/m^3^.

Daily numbers of emergency hospital visits, with HD, respiratory diseases and stroke as primary diagnoses were counted according to gender and age groups, and time-series plots were made (S1 Fig) as well as time-series plots for H_2_S concentrations (Fig 2). We used a generalized linear model (GLM) assuming Poisson distribution of outcome measures to estimate the association between short-term daily exposures to H_2_S. This method was chosen since hospital admissions and ED visits are a discrete counting event [33] and the method is often used to investigate short-term associations of environmental exposures with various health outcomes [34,35].

Daily numbers of emergency hospital visits were the dependent variable. Separate analyses were performed for HD, respiratory diseases, and stroke as primary diagnosis. Modelled H_2_S concentrations at patient’s residence were selected as independent variables, classified as different percentiles of H_2_S exposure (50%, 60%, 70%, 80%, 85%, 90% and 95%). Population data was used as offset to account for population size and demographic composition (age and gender) in each section. To control for seasonality and long-term trends in outcome measures, models were adjusted for day-of-week and basic spline with 8 degrees of freedom as it gave the best model fit. The number of degrees of freedom is essential to minimize the autocorrelation in the residuals and to account for seasonal trends in outcome measures [35]. Here, a small number of degrees of freedom was chosen since long-term seasonal trends in number of emergency hospital visits did not seem apparent [34].

A number of models were tested. First, we ran a crude analysis testing the association between H_2_S (classified as different percentiles of H_2_S exposure) exposure and outcome while adjusting for seasonality (splines) only. Secondly, fully adjusted models were distributed lag models [35] and were adjusted for seasonality (splines), gender, age group, traffic exposure zone, distance from Hellisheidi power plant, and same-day average temperature using different percentiles of H_2_S exposure. Measured concentrations of traffic-related pollution (NO_2_, O_3_, PM_10_, and SO_2_) were tested in the model and did not modify the association, and were thus omitted. Also, potential autocorrelation was avoided by adjusting the model with the number of each outcome measure at lag 1 (previous day). Thirdly, H_2_S concentrations at different sections of the Reykjavik capital area were introduced to fully adjusted models as a continuous variable giving results for an increase of 7μg/m^3^ in H_2_S concentrations. Fourthly, dose-response trends were analysed through different percentiles of H_2_S exposure levels (50%, 60%, 70%, 80%, 85%, 90%, and 95%) using GLM analysis. Due to dependency of RR estimates within each lag, all H_2_S exposure levels were introduced in the model at the same time. Lag days from 0 to 4 were considered in each model. Backwards selection of adjustment variables showed that season, humidity and lags 5 to 7 did not significantly affect the results and were therefore not included in fully adjusted models. Residual analysis and graphical assessment of autocorrelation and spline functions indicated that modelling assumptions were rational.

The analysis yielded risk ratio (RR) and 95% confidence interval (CI) for each lag structure. Here, the focus will be on results for H_2_S concentrations exceeding 7.00 μg/m^3^ (85% exposure level) and emergency hospital visits with HD, respiratory disease, or stroke as primary diagnosis (other results are shown in Supporting Information). Results with p-value less than 0.05 were considered statistically significant.

Data were prepared and statistical analyses were performed using R statistical software, version 3.1.3 [36].

The study and use of the data were approved by Bioethics Committee (VSNb2010120017/03.7), the Data Protection Agency (2010121176AT/), and the Hospital ethics board (Letter dated 2010/12/22).

## Results

The mid-year population of adults (18 years and older) in the Reykjavik capital area was 151095 in year 2010 [24]. During the seven and a half year period (2738 days), there were 13383 patients with a total of 32961 emergency hospital visits to LUH (Table 1), where the proportion of male visits was 56.8%. The average number of daily emergency hospital visits over the study period was 12.0 with a range of 0-32 visits per day (Table 1). Most emergency hospital visits were with HD as primary diagnosis, followed by respiratory diseases. The average number of daily emergency hospital visits with stroke diagnosis was approximately 2.35. Median age of all patients was 73 years. Mean age of patients was 69.9 years with the highest mean age of female HD patients (74.8 years). Patients with respiratory diseases as a primary diagnosis had the youngest mean age (66.5 years). Female patients with emergency hospital visit were on average 3.8 years older than males.

**Table 1:** Descriptive statistics of daily emergency hospital visits to Landspitali University Hospital, according to primary diagnosis, during 1 January, 2007 to 30 June, 2014.

	No. of visits n (%)	Visits/day Mean (±SD)	Visits/day Range	Visits/day Median	Percentiles	
Emergency hospital patients					25^th^	75^th^
All primary diagnosis	32961 (100)	12.04 (4.86)	1–32	12	8	15
Females	14224 (43.2)	5.28 (2.70)	0–16	5	3	7
Males	18737 (56.8)	6.98 (3.41)	0–21	7	4	9
Older (**≥**73yr)	15885 (48.19)	5.88 (2.92)	0–20	6	4	8
Younger (<73yr)	17076 (51.81)	6.30 (3.17)	0–20	6	4	8
Heart diseases as primary diagnosis	20529 (62.3)	7.54 (6.65)	0–23	7	5	10
Females	7400	3.02 (1.77)	0–12	3	2	4
Males	13129	4.94 (2.72)	0–19	5	3	7
Older (**≥**73yr)	9868	3.80 (2,13)	0–16	3	2	5
Younger (<73yr)	10661	4.11 (2.43)	0–16	4	2	6
Respiratory diseases as primary diagnosis	7438 (22.6)	3.00 (1.80)	0–13	3	2	4
Females	4515	2.18 (1.32)	0–10	2	1	3
Males	2923	1.74 (0.98)	0–7	1	1	2
Older (**≥**73yr)	3198	1.83 (1.04)	0–7	2	1	2
Younger (<73yr)	4240	2.12 (1.26)	0–8	2	1	3
Stroke as primary diagnosis	4994 (15.2)	2.35 (1.46)	0–11	2	1	3
Females	2309	1.65 (0.90)	0–6	1	1	2
Males	2685	1.81 (1.07)	0–8	1	1	2
Older (**≥**73yr)	2819	1.84 (1.06)	0–9	2	1	2
Younger (<73yr)	2175	1.66 (0.93)	0–7	1	1	2

The modelled 24-hour mean concentrations within each section (A-E) are shown in Table 2 and Fig 2. Overall, 75% of all modelled values of 24-hour H_2_S concentrations were lower than 5 μg/m^3^. The mean 24-hour H_2_S concentration was highest in section D with an average concentration of 4.04 μg/m^3^, and lowest in section A (3.02 μg/m^3^). The highest 24-hour H_2_S concentration was 69.5 μg/m^3^ in section C (Fig 2), and in section A, the highest concentration was 37.0 μg/m^3^. The number of 24-hour concentrations exceeding the different percentiles and the percentiles’ lower limits in μg/m^3^ within each section are shown in Table 3. The correlation of 24-hour H_2_S concentration between sections in the Reykjavik capital area ranged from 0.05 between sections A and E up to 0.80 between sections D and E (Table 2).

**Table 2:** Descriptive statistics of modelled daily 24-hour concentrations of H_2_S during study period in each section of the Reykjavik capital area, daily count of higher concentration in each section and percentiles, as well as Spearman´s correlation of daily 24-hour concentrations of H_2_S between sections.

During study period	Section A	Section B	Section C	Section D	Section E
Modelled days in study period	2738	2738	2738	2738	2738
Mean concentration (μg/m^3^) (±SD)	3.02 (4.05)	3.53 (5.34)	3.79 (5.96)	4.04 (6.83)	3.89 (7.10)
Range (μg/m^3^)	0–37.0	0–48.1	0–69.5	0–68.2	0–66.9
Interquartile range (μg/m^3^) (0.25, 0.75)	0.0, 4.8	0.1, 4.5	0.2, 4.9	0.2, 4.9	0.2, 4.6
**Number of high concentrations within section**					
**Lower limits of percentiles**					
50% (≥2.46 μg/m^3^)	498	986	1250	862	266
60% (≥3.16 μg/m^3^)	436	833	1091	743	235
70% (≥4.14 μg/m^3^)	345	632	903	567	188
80% (≥5.74 μg/m^3^)	241	461	646	393	135
85% (≥7.00 μg/m^3^)	177	362	533	321	106
90% (≥8.80 μg/m^3^)	124	257	395	263	75
95% (≥11.68 μg/m^3^)	58	158	259	171	49
**Spearman´s correlation**					
Section A	1.00				
Section B	0.67	1.00			
Section C	0.39	0.73	1.00		
Section D	0.17	0.37	0.75	1.00	
Section E	0.05	0.18	0.46	0.80	1.00

**Table 3:** Number of emergency hospital visits to Landspitali University Hospital, in each modelled section of the Reykjavik capital area, and in higher percentiles of H_2_S concentrations during 1 January, 2007 to 30 June, 2014.

Lower limits of percentiles	All sections	Section A	Section B	Section C	Section D	Section E
50% (≥2.46 μg/m^3^)	14157	835	2813	8130	1985	394
60% (≥3.16 μg/m^3^)	12137	730	2381	6961	1714	351
70% (≥4.14 μg/m^3^)	9614	585	1802	5669	1284	274
80% (≥5.74 μg/m^3^)	6853	397	1340	4031	878	207
85% (≥7.00 μg/m^3^)	5596	288	1067	3347	726	168
90% (≥8.80 μg/m^3^)	4089	199	746	2453	578	113
95% (≥11.68 μg/m^3^)	2616	93	463	1604	381	75
Total visits (%)	32961 (100)	1895 (5.7)	6678 (20.3)	18934 (57.4)	4502 (13.7)	952 (2.9)
Total inhabitants (%)	151095 (100)	11868 (7.9)	29168 (19.3)	83703 (55.4)	20220 (13.4)	6136 (4.1)

The crude analysis for the association of H_2_S concentrations exceeding 7.00 μg/m^3^ and HD, respiratory diseases and stroke as primary diagnosis when adjusting only for seasonality (splines) is shown in S1 Table. An overall increase in RRs was seen for every outcome measure at every lag, though the confidence intervals (CI) were wide and included unity. In S2 Table shows the RR for every 7.00 μg/m^3^ increase in H_2_S concentration (introduced as continuous variable) for HD diseases, respiratory diseases, and stroke as primary diagnosis from fully adjusted models. The only CI not including unity was for stroke as primary diagnosis at lag 2.

**Fig 3:**
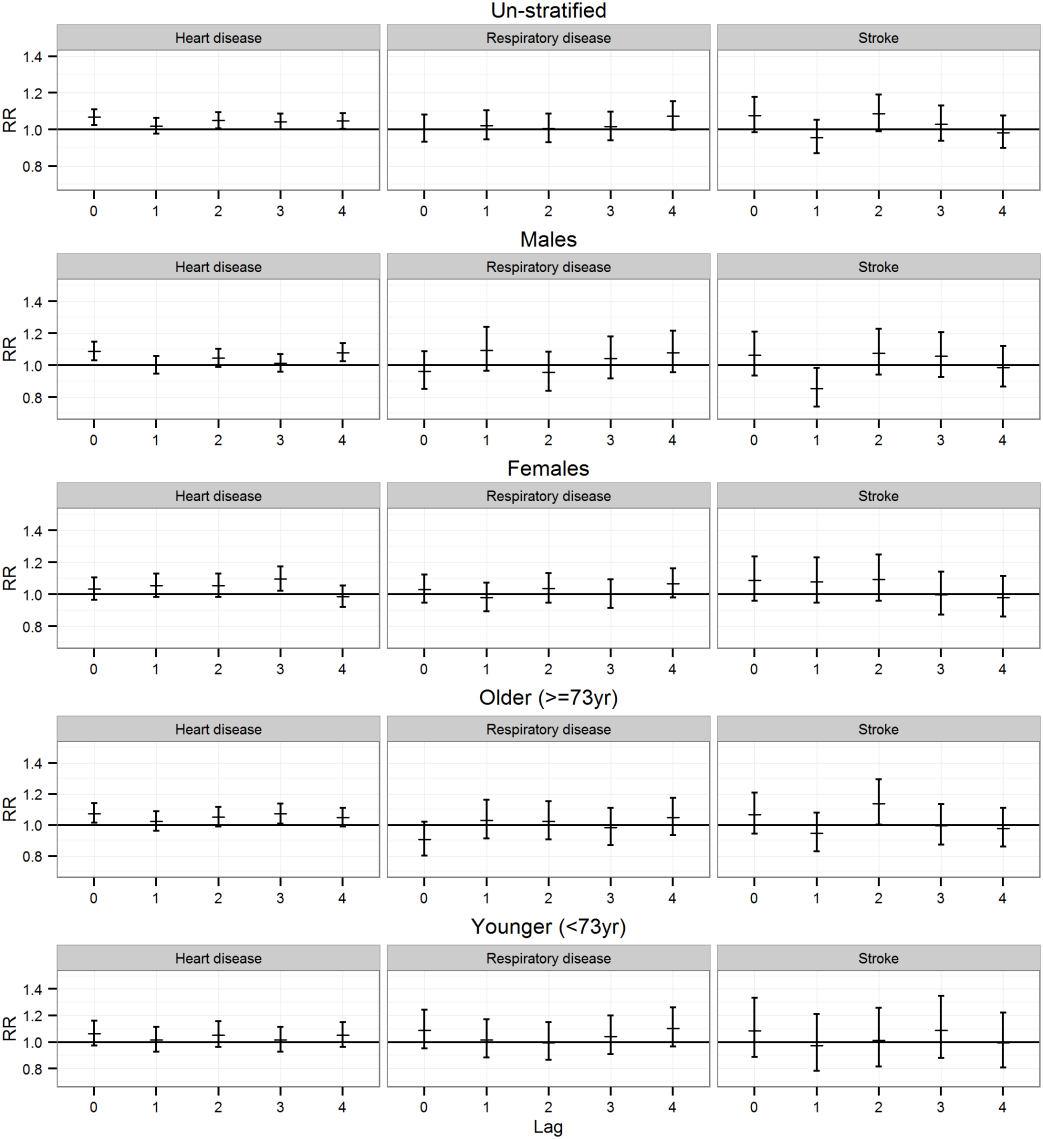
Associations between daily emergency hospital visits with heart diseases, respiratory diseases, and stroke as primary diagnosis and H_2_S concentrations exceeding 7.00 μg/m^3^ in fully adjusted models for lags 0–4, un-stratified, and gender and age stratification.

In the fully adjusted analysis in un-stratified models, statistically significant associations were found between H_2_S concentrations exceeding 7.00 μg/m^3^ and increases in emergency hospital visits with HD as primary diagnosis at lags 0, 2, and 4. There were also increases at lags 1 and, 3, though CIs included unity (Fig 3). Trend analyses between different levels of exposure (from 50 to 95 percentiles) gave p<0.05 at lags 0 and 2, indicating a positive dose-response association (Table 4). P-values for trend analysis at lag 4 were also <0.05 for a negative dose-response (Table 4). When analysis was stratified by gender, associations were found among males and between H_2_S concentrations exceeding 7.00 μg/m^3^ and HD at lags 0 and 4 (Fig 3); however the trend analysis between different exposure levels was not significant at any lag. Among females, an association was found at lag 3 (Fig 3). Also among females, p-values for trend analysis between different exposure levels indicated a positive dose-response association at lags 0 and 2 but a negative dose-response at lag 4 (Table 4). Analyses stratified by age showed associations between H_2_S concentrations exceeding 7.00 μg/m^3^ among those 73 years and older at lags 0 and 3, whereas CIs did not include unity (Table 4). Additionally, p-values for trend analysis were <0.05 at lags 0, 2, and 3, indicating a positive dose-response association (Table 4).

**Table 4:** Associations between daily emergency hospital visits with heart diseases as primary diagnosis and different percentiles of H_2_S exposure in fully adjusted models for lags 0–4, un-stratified, and gender and age stratification.

	50% (≥2.46 μg/m^3^)		60% (≥3.16 μg/m^3^)		70% (≥4.14 μg/m^3^)		80% (≥5.74 μg/m^3^)		85% (≥7.00 μg/m^3^)		90% (≥8.80 μg/m^3^)		95% (≥11.68 μg/m^3^)		
Lag	RR	95% CI	RR	95% CI	RR	95% CI	RR	95% CI	RR	95% CI	RR	95% CI	RR	95% CI	p-trend
**Un-stratified**^a^															
**0**	1.007	1.004, 1.009	1.056	1.023, 1.091	1.048	1.013, 1.084	1.068	1.028, 1.109	1.067	1.024, 1.111	1.071	1.022, 1.121	1.059	1.001, 1.122	0.0038
**1**	1.008	1.005, 1.010	1.073	1.037, 1.109	1.056	1.019, 1.094	1.031	0.991, 1.072	1.019	0.976, 1.064	1.011	0.962, 1.061	0.986	0.928, 1.049	0.1927
**2**	1.006	1.003, 1.009	1.043	1.009, 1.079	1.047	1.011, 1.085	1.057	1.017, 1.100	1.049	1.005, 1.095	1.045	0.995, 1.098	1.062	1.001, 1.128	0.0027
**3**	1.005	1.003, 1.008	1.053	1.019, 1.089	1.043	1.007, 1.081	1.034	0.994, 1.075	1.042	0.999, 1.087	1.012	0.964, 1.063	1.010	0.952, 1.072	0.7116
**4**	1.060	1.027, 1.094	1.050	1.016, 1.084	1.043	1.008, 1.079	1.037	0.998, 1.077	1.046	1.004, 1.089	1.053	1.005, 1.103	1.020	0.962, 1.081	0.0483
**Gender stratification**^b^															
**Males**															
**0**	1.010	1.006, 1.013	1.078	1.034, 1.124	1.072	1.026, 1.121	1.091	1.039, 1.145	1.087	1.032, 1.146	1.080	1.018, 1.146	1.067	0.992, 1.148	0.0627
**1**	1.007	1.003, 1.010	1.066	1.021, 1.113	1.061	1.014, 1.111	1.023	0.973, 1.077	1.000	0.946, 1.057	1.017	0.955, 1.083	1.017	0.940, 1.100	0.4006
**2**	1.006	1.003, 1.009	1.052	1.007, 1.099	1.043	0.996, 1.092	1.053	1.001, 1.107	1.045	0.989, 1.104	1.042	0.978, 1.110	1.033	0.955, 1.116	0.3038
**3**	1.005	1.002, 1.008	1.034	0.991, 1.080	1.028	0.982, 1.076	1.018	0.968, 1.071	1.012	0.958, 1.069	0.978	0.918, 1.042	0.983	0.910, 1.062	0.0879
**4**	1.085	1.042, 1.130	1.070	1.027, 1.116	1.057	1.012, 1.105	1.066	1.015, 1.119	1.080	1.025, 1.138	1.089	1.026, 1.156	1.065	0.989, 1.147	0.9065
**Females**															
**0**	1.002	0.998, 1.006	1.019	0.967, 1.075	1.006	0.951, 1.064	1.029	0.967, 1.095	1.033	0.965, 1.105	1.055	0.978, 1.139	1.047	0.953, 1.151	0.0000
**1**	1.010	1.005, 1.014	1.087	1.029, 1.148	1.046	0.987, 1.109	1.045	0.980, 1.114	1.054	0.983, 1.130	1.000	0.923, 1.084	0.933	0.843, 1.033	0.1910
**2**	1.006	1.002, 1.010	1.025	0.970, 1.083	1.052	0.993, 1.115	1.061	0.996, 1.131	1.054	0.983, 1.129	1.047	0.967, 1.134	1.113	1.011, 1.225	0.0004
**3**	1.006	1.001, 1.010	1.086	1.029, 1.147	1.069	1.009, 1.132	1.060	0.995, 1.130	1.096	1.023, 1.174	1.076	0.994, 1.163	1.060	0.963, 1.166	0.1761
**4**	1.015	0.964, 1.069	1.013	0.960, 1.068	1.018	0.963, 1.077	0.987	0.928, 1.050	0.985	0.921, 1.054	0.987	0.914, 1.066	0.940	0.854, 1.034	0.0010
**Age stratification**^c^															
**Older (≥73yr)**															
**0**	1.008	1.004, 1.011	1.062	1.014, 1.113	1.059	1.008, 1.112	1.069	1.012, 1.129	1.075	1.014, 1.140	1.080	1.011, 1.154	1.096	1.010, 1.189	0.0000
**1**	1.010	1.006, 1.014	1.093	1.042, 1.147	1.053	1.001, 1.109	1.037	0.980, 1.097	1.024	0.963, 1.090	1.026	0.956, 1.100	0.993	0.909, 1.085	0.2710
**2**	1.006	1.003, 1.010	1.023	0.975, 1.074	1.048	0.996, 1.103	1.056	0.998, 1.117	1.052	0.990, 1.118	1.045	0.974, 1.121	1.057	0.97, 1.152	0.0000
**3**	1.007	1.003, 1.011	1.058	1.008, 1.110	1.042	0.991, 1.096	1.061	1.003, 1.122	1.072	1.009, 1.139	1.077	1.006, 1.154	1.080	0.993, 1.174	0.0000
**4**	1.036	0.99, 1.0840	1.049	1.001, 1.099	1.054	1.004, 1.107	1.034	0.979, 1.091	1.049	0.989, 1.112	1.069	1.000, 1.142	1.006	0.926, 1.093	0.7273
**Younger (<73yr)**															
**0**	1.007	1.001, 1.012	1.056	0.985, 1.133	1.042	0.968, 1.122	1.073	0.989, 1.163	1.062	0.973, 1.160	1.064	0.963, 1.175	1.026	0.906, 1.162	0.2849
**1**	1.006	1.000, 1.012	1.056	0.982, 1.135	1.064	0.986, 1.148	1.026	0.943, 1.117	1.016	0.927, 1.114	1.002	0.902, 1.113	0.988	0.866, 1.127	0.2173
**2**	1.006	1.000, 1.012	1.070	0.996, 1.151	1.050	0.973, 1.134	1.061	0.976, 1.154	1.054	0.962, 1.155	1.047	0.943, 1.163	1.076	0.946, 1.224	0.0926
**3**	1.004	0.999, 1.010	1.059	0.985, 1.138	1.052	0.975, 1.135	1.011	0.929, 1.100	1.017	0.928, 1.115	0.953	0.856, 1.060	0.949	0.833, 1.081	0.0541
**4**	1.084	1.013, 1.161	1.049	0.978, 1.125	1.030	0.957, 1.108	1.049	0.968, 1.138	1.051	0.962, 1.148	1.052	0.950, 1.164	1.047	0.924, 1.186	0.2137

^a^Un-stratified models were adjusted for gender, age group, season, day-of-week, distance from Hellisheidi power plant, traffic exposure zone and temperature.

^b^Gender stratified models were adjusted for age group, season, day-of-week, distance from Hellisheidi power plant, traffic exposure zone and temperature.

^c^Age stratified models were adjusted for gender, season, day-of-week, distance from Hellisheidi power plant, traffic exposure zone and temperature

The RRs for the association between H_2_S at different percentiles and emergency hospital visits with respiratory diseases as primary diagnosis is shown in Table 5. In fully adjusted analysis, both un-stratified and stratified by gender and age, models for H_2_S concentrations exceeding 7.00 μg/m^3^ were not statistically associated with an increase or decrease in emergency hospital visits with respiratory diagnosis at any lag (Table 5). On the other hand, some trends through different levels of exposure (from 50 to 95 percentiles) were significant at lag 0, and two other lags in the un-stratified analysis, and in male and the older strata, indicating a negative dose-response association (Table 5).

**Table 5:** Associations between daily emergency hospital visits with respiratory diseases as primary diagnosis and different percentiles of H_2_S exposure in fully adjusted models for lags 0–4, un-stratified, and gender and age stratification.

	50% (≥2.46 μg/m^3^)		60% (≥3.16 μg/m^3^)		70% (≥4.14 μg/m^3^)		80% (≥5.74 μg/m^3^)		85% (≥7.00 μg/m^3^)		90% (≥8.80 μg/m^3^)		95% (≥11.68 μg/m^3^)		
	RR	95% CI	RR	95% CI	RR	95% CI	RR	95% CI	RR	95% CI	RR	95% CI	RR	95% CI	p-trend
**Un-stratified**^a^															
**0**	1.009	1.005, 1.014	1.059	0.999, 1.123	1.040	0.978, 1.106	1.012	0.945, 1.083	1.003	0.931, 1.081	0.979	0.899, 1.065	0.980	0.882, 1.088	0.0340
**1**	1.004	0.999, 1.009	1.038	0.977, 1.103	1.011	0.948, 1.078	1.031	0.961, 1.107	1.022	0.946, 1.105	1.031	0.944, 1.126	1.003	0.898, 1.121	0.9040
**2**	1.005	1.001, 1.010	1.024	0.964, 1.088	1.045	0.980, 1.114	1.047	0.976, 1.123	1.005	0.930, 1.086	0.973	0.890, 1.064	0.951	0.851, 1.063	0.1702
**3**	1.006	1.002, 1.011	1.055	0.993, 1.120	1.026	0.962, 1.093	1.004	0.936, 1.077	1.015	0.940, 1.096	1.001	0.917, 1.093	0.950	0.851, 1.059	0.0721
**4**	1.101	1.040, 1.165	1.105	1.043, 1.172	1.096	1.031, 1.164	1.108	1.037, 1.185	1.073	0.998, 1.155	1.085	0.999, 1.179	1.106	0.999, 1.225	0.4642
**Gender stratification**^b^															
**Males**															
**0**	1.007	0.999, 1.014	1.032	0.938, 1.136	1.026	0.928, 1.135	0.988	0.882, 1.105	0.963	0.851, 1.089	0.950	0.825, 1.093	0.944	0.793, 1.123	0.0003
**1**	1.000	0.993, 1.008	1.053	0.953, 1.163	1.015	0.914, 1.128	1.075	0.958, 1.207	1.094	0.965, 1.240	1.066	0.923, 1.230	1.021	0.851, 1.224	0.2308
**2**	1.006	0.999, 1.014	1.040	0.942, 1.149	1.028	0.926, 1.142	0.999	0.889, 1.123	0.955	0.841, 1.085	0.939	0.811, 1.089	0.922	0.768, 1.108	0.0009
**3**	1.006	0.999, 1.014	1.046	0.948, 1.154	1.039	0.937, 1.153	1.000	0.891, 1.123	1.042	0.919, 1.181	1.002	0.867, 1.158	0.971	0.813, 1.160	0.2599
**4**	1.112	1.013, 1.221	1.143	1.039, 1.256	1.138	1.031, 1.257	1.152	1.033, 1.284	1.078	0.956, 1.216	1.065	0.928, 1.221	1.132	0.959, 1.336	0.4502
**Females**															
**0**	1.011	1.005, 1.016	1.079	1.008, 1.154	1.051	0.978, 1.128	1.028	0.950, 1.113	1.031	0.946, 1.124	0.996	0.903, 1.098	1.003	0.888, 1.132	0.2065
**1**	1.006	1.001, 1.012	1.028	0.958, 1.103	1.009	0.936, 1.087	1.003	0.923, 1.089	0.980	0.895, 1.073	1.012	0.914, 1.122	0.994	0.874, 1.131	0.1771
**2**	1.005	1.000, 1.010	1.014	0.945, 1.089	1.056	0.980, 1.138	1.077	0.993, 1.169	1.037	0.948, 1.134	0.991	0.894, 1.099	0.967	0.850, 1.099	0.6453
**3**	1.006	1.001, 1.012	1.061	0.990, 1.138	1.017	0.944, 1.095	1.007	0.928, 1.093	1.001	0.915, 1.095	1.004	0.907, 1.112	0.936	0.824, 1.064	0.0572
**4**	1.093	1.023, 1.168	1.080	1.010, 1.156	1.066	0.993, 1.145	1.079	0.998, 1.166	1.067	0.980, 1.162	1.098	0.997, 1.208	1.090	0.968, 1.228	0.8976
**Age stratification**^c^															
**Older (≥73yr)**															
**0**	1.003	0.996, 1.010	0.971	0.885, 1.064	0.934	0.847, 1.030	0.916	0.821, 1.022	0.906	0.804, 1.022	0.862	0.751, 0.990	0.868	0.732, 1.029	0.0000
**1**	1.005	0.998, 1.012	1.098	0.999, 1.207	1.035	0.936, 1.145	1.050	0.940, 1.173	1.029	0.912, 1.163	1.059	0.922, 1.215	1.014	0.852, 1.207	0.8427
**2**	1.005	0.998, 1.013	1.003	0.912, 1.103	1.055	0.954, 1.166	1.057	0.946, 1.180	1.023	0.906, 1.154	0.994	0.864, 1.143	0.983	0.827, 1.168	0.7260
**3**	1.006	0.999, 1.013	1.037	0.943, 1.139	1.014	0.918, 1.120	0.974	0.872, 1.088	0.983	0.871, 1.109	0.894	0.776, 1.030	0.895	0.753, 1.064	0.0013
**4**	1.059	0.969, 1.158	1.063	0.970, 1.164	1.029	0.934, 1.133	1.064	0.957, 1.183	1.048	0.934, 1.176	1.106	0.971, 1.259	1.131	0.966, 1.325	0.0881
**Younger (<73yr)**															
**0**	1.015	1.006, 1.023	1.143	1.028, 1.271	1.137	1.017, 1.271	1.093	0.966, 1.237	1.087	0.951, 1.243	1.079	0.927, 1.255	1.079	0.895, 1.300	0.8132
**1**	1.004	0.995, 1.013	0.992	0.888, 1.109	0.994	0.884, 1.118	1.019	0.895, 1.160	1.018	0.884, 1.172	1.010	0.860, 1.187	0.990	0.809, 1.212	0.6274
**2**	1.006	0.998, 1.015	1.046	0.936, 1.169	1.042	0.927, 1.172	1.041	0.915, 1.184	0.996	0.865, 1.148	0.960	0.815, 1.131	0.932	0.759, 1.143	0.0543
**3**	1.007	0.998, 1.015	1.075	0.963, 1.201	1.039	0.925, 1.168	1.034	0.909, 1.176	1.043	0.907, 1.200	1.094	0.934, 1.281	0.995	0.816, 1.213	0.8912
**4**	1.138	1.025, 1.264	1.144	1.029, 1.272	1.152	1.031, 1.287	1.151	1.019, 1.299	1.103	0.966, 1.261	1.081	0.929, 1.258	1.099	0.912, 1.326	0.0274

^a^Models adjusted for gender, age group, season, day-of-week, distance from Hellisheidi power plant, traffic exposure zone and temperature.

^b^Models adjusted for age group, season, day-of-week, distance from Hellisheidi power plant, traffic exposure zone and temperature.

^c^Models adjusted for gender, season, day-of-week, distance from Hellisheidi power plant, traffic exposure zone and temperature.

The RRs for the association between H_2_S at different percentiles and emergency hospital visits with stroke as primary diagnosis are shown in Table 6. In the fully adjusted analysis in un-stratified models, non-significant associations between H_2_S concentrations exceeding 7.00 μg/m^3^ and emergency hospital visits with stroke were found (Table 6). When analysis was stratified by age, a statistically significant association was found at lag 2 among those 73 years and older (Fig 3, and in Table 6). In the trend analyses through different levels of exposure (from 50 to 95 percentiles), statistically significant positive association was found at lag 0 in un-stratified, males, and the older stratum, and statistically significant negative association at lag 1 in the same stratum, indicating dose-response manner of association (Table 6).

**Table 6:** Association between daily emergency hospital visits with stroke as primary diagnosis and different percentiles of H_2_S exposure in fully adjusted models for lags 0–4, un-stratified, and gender and age stratification.

	50% (≥2.46 μg/m^3^)		60% (≥3.16 μg/m^3^)		70% (≥4.14 μg/m^3^)		80% (≥5.74 μg/m^3^)		85% (≥7.00 μg/m^3^)		90% (≥8.80 μg/m^3^)		95% (≥11.68 μg/m^3^)		
	RR	95% CI	RR	95% CI	RR	95% CI	RR	95% CI	RR	95% CI	RR	95% CI	RR	95% CI	p-trend
**Un-stratified**^a^															
**0**	1.010	1.005, 1.016	1.074	1.000, 1.153	1.058	0.981, 1.140	1.049	0.965, 1.140	1.076	0.984, 1.178	1.081	0.977, 1.197	1.126	0.996, 1.274	0.0038
**1**	1.004	0.998, 1.009	1.016	0.943, 1.093	1.008	0.932, 1.089	0.966	0.885, 1.054	0.957	0.870, 1.053	0.956	0.858, 1.066	0.983	0.860, 1.124	0.0086
**2**	1.009	1.003, 1.014	1.109	1.030, 1.193	1.170	1.083, 1.263	1.130	1.038, 1.231	1.086	0.989, 1.192	1.145	1.031, 1.272	1.105	0.970, 1.258	0.2032
**3**	1.007	1.001, 1.013	1.036	0.963, 1.115	1.004	0.929, 1.085	1.044	0.958, 1.137	1.029	0.937, 1.130	1.019	0.916, 1.135	0.984	0.861, 1.124	0.7005
**4**	1.018	0.950, 1.092	1.029	0.958, 1.104	1.036	0.961, 1.116	0.987	0.908, 1.073	0.983	0.897, 1.077	0.950	0.855, 1.056	0.894	0.783, 1.021	0.0013
**Gender stratification**^b^															
**Males**															
**0**	1.011	1.003, 1.018	1.069	0.967, 1.182	1.030	0.926, 1.145	1.031	0.915, 1.160	1.065	0.937, 1.211	1.105	0.957, 1.275	1.152	0.968, 1.372	0.0104
**1**	0.999	0.991, 1.007	1.000	0.901, 1.110	1.001	0.896, 1.118	0.899	0.792, 1.019	0.854	0.743, 0.982	0.860	0.735, 1.008	0.898	0.738, 1.092	0.0002
**2**	1.008	1.000, 1.016	1.071	0.966, 1.189	1.114	0.998, 1.242	1.101	0.976, 1.243	1.076	0.942, 1.228	1.158	0.997, 1.344	1.076	0.892, 1.298	0.0654
**3**	1.009	1.001, 1.017	1.068	0.963, 1.185	1.022	0.916, 1.141	1.072	0.950, 1.209	1.058	0.928, 1.207	1.039	0.893, 1.209	1.016	0.841, 1.227	0.7996
**4**	1.021	0.926, 1.127	1.004	0.908, 1.111	1.045	0.940, 1.161	0.995	0.884, 1.119	0.985	0.866, 1.121	0.886	0.761, 1.031	0.789	0.648, 0.961	0.0148
**Females**															
**0**	1.010	1.002, 1.018	1.079	0.976, 1.194	1.091	0.981, 1.213	1.071	0.953, 1.204	1.089	0.960, 1.236	1.054	0.913, 1.218	1.098	0.922, 1.307	0.1021
**1**	1.008	1.000, 1.017	1.035	0.932, 1.149	1.016	0.910, 1.134	1.043	0.924, 1.178	1.079	0.946, 1.231	1.071	0.923, 1.244	1.080	0.898, 1.299	0.0000
**2**	1.009	1.001, 1.017	1.152	1.038, 1.278	1.234	1.107, 1.376	1.162	1.031, 1.309	1.095	0.960, 1.248	1.129	0.974, 1.310	1.134	0.946, 1.358	0.4811
**3**	1.005	0.997, 1.013	1.002	0.902, 1.112	0.984	0.881, 1.098	1.014	0.897, 1.145	0.998	0.873, 1.141	0.997	0.856, 1.161	0.949	0.787, 1.145	0.1973
**4**	1.014	0.918, 1.120	1.055	0.954, 1.167	1.024	0.921, 1.139	0.977	0.868, 1.101	0.980	0.860, 1.116	1.025	0.885, 1.186	1.016	0.849, 1.216	0.3792
**Age stratification**^c^															
**Older (≥73yr)**															
**0**	1.010	1.002, 1.017	1.068	0.968, 1.178	1.047	0.944, 1.162	1.038	0.925, 1.165	1.068	0.943, 1.210	1.076	0.935, 1.238	1.151	0.972, 1.363	0.0136
**1**	1.007	0.999, 1.015	1.031	0.931, 1.141	0.984	0.883, 1.096	0.966	0.857, 1.091	0.947	0.829, 1.081	0.963	0.829, 1.118	0.975	0.811, 1.173	0.0042
**2**	1.011	1.003, 1.019	1.107	1.000, 1.225	1.228	1.105, 1.365	1.165	1.036, 1.310	1.138	1.002, 1.294	1.191	1.032, 1.376	1.242	1.043, 1.479	0.0059
**3**	1.006	0.998, 1.013	1.025	0.926, 1.135	0.982	0.882, 1.093	1.031	0.915, 1.161	0.996	0.874, 1.135	1.000	0.862, 1.161	0.899	0.745, 1.084	0.1683
**4**	1.030	0.935, 1.135	1.037	0.940, 1.144	1.056	0.952, 1.171	0.952	0.847, 1.070	0.978	0.861, 1.110	0.949	0.820, 1.098	0.905	0.753, 1.088	0.0005
**Younger (<73yr)**															
**0**	1.011	0.999, 1.024	1.092	0.928, 1.284	1.085	0.914, 1.286	1.068	0.883, 1.291	1.086	0.885, 1.333	1.090	0.864, 1.375	1.088	0.817, 1.448	0.0591
**1**	0.999	0.986, 1.013	0.998	0.843, 1.183	1.048	0.877, 1.252	0.962	0.787, 1.175	0.973	0.782, 1.211	0.953	0.742, 1.223	1.014	0.743, 1.382	0.4534
**2**	1.005	0.992, 1.018	1.115	0.942, 1.320	1.085	0.908, 1.296	1.074	0.883, 1.308	1.012	0.815, 1.257	1.082	0.848, 1.381	0.911	0.665, 1.246	0.3775
**3**	1.010	0.997, 1.024	1.061	0.897, 1.256	1.044	0.875, 1.247	1.077	0.886, 1.309	1.089	0.880, 1.347	1.050	0.822, 1.341	1.140	0.848, 1.533	0.0064
**4**	1.005	0.858, 1.178	1.015	0.862, 1.195	1.006	0.847, 1.194	1.044	0.865, 1.261	0.993	0.806, 1.223	0.963	0.757, 1.225	0.891	0.658, 1.208	0.0996

^a^Models adjusted for gender, age group, season, day-of-week, distance from Hellisheidi power plant, traffic exposure zone and temperature.

^b^Models adjusted for age group, season, day-of-week, distance from Hellisheidi power plant, traffic exposure zone and temperature.

^c^Models adjusted for gender, season, day-of-week, distance from Hellisheidi power plant, traffic exposure zone and temperature

## Discussion

The present study showed an association between hospital admission and emergency department visits with heart disease (HD) as primary diagnosis and H_2_S concentrations exceeding 7.00 μg/m^3^ occurring the same day, more pronounced among males and those 73 years and older than among females and younger individuals. The associations were also seen with delay of two and four days. Same-day associations were gradually increasing through higher percentiles of exposure in a dose-response manner of relationship. These results are in concordance with previous studies on possible short-term effects, which were based on hospital records [19–22]. The present results are also supported by a study on association of increased general mortality and H_2_S exposure, conducted in the Reykjavik capital area [16], and by another study in the same setting on increased dispensing of anti-asthma drugs and H_2_S levels [9]. On the other hand, in the medical literature, it has been discussed that low-level H_2_S intracellularly may reduce vasoconstriction and promote cardiovascular health [37,38]. Contrary, H_2_S exposure in healthy human volunteers as low as 5 ppm were associated with a shift from aerobic to anaerobic metabolism [39], however, whether this has relevance in the present study is merely speculative.

The chosen time unit in the present study for H_2_S concentrations and for emergency hospital visits was 24 hours. The argument for the definition of the 24-hour H_2_S μg/m^3^ was that the distribution was skewed, as shown in Table 2 and Fig 2 and in S2 and S3 Figs, where only one or two times per month did the H_2_S concentration exceed the odour limit of 7.00 H_2_S μg/m^3^ over two consecutive days. The rationale for the definition of sum of admissions and visits per 24 hours (and not for example hourly admissions and visits) is the diurnal distribution, where hospital visits accumulate during office hours (shown in S4 Fig).

In the present study, the pattern seen in increased risk of emergency hospital visits associated with increased percentiles of H_2_S concentrations is compatible with a harvesting effect [40]; namely, there is a positive dose-response association at lag 0 and lag 2, and negative dose-response association at lag 4 through the un-stratified analysis, and among females. The hypothesized harvesting effect [40] is not very obvious from the lag analyses. However, in the calculation on H_2_S concentrations exceeding 7.00 μg/m^3^, the risk estimation was highest for lag 0 and lowest for lag 1 in un-stratified, male stratum, and both age strata, giving further support for harvesting effect and the possibility of short-term effects of H_2_S exposure. Considering the intermittent H_2_S exposure, however, it is not possible to evaluate long-term health effects of H_2_S exposure in the present study setting, and for that a reference group from an unexposed region may be needed. The outcome in the present study, emergency hospital visits with HD as primary diagnosis, brings to mind the many known risks and causal factors for these diseases. However, respecting other causal effects, these should not preclude the possible short-term effect of an environmental pollution such as H_2_S. The present results are not in contradiction with the studies from Rotorua using a cross-sectional approach, which have not found associations between long-term H_2_S exposure and asthma and chronic obstructive pulmonary diseases [10,23]. Here, emergency hospital visits with respiratory diseases were not associated with H_2_S exposure; however, the study had limited power to detect such possible association.

Backwards selection showed that the traffic exposure zone (distance from main roads) was a better fit in the fully adjusted analysis rather than the measured traffic-related pollution (NO_2_, O_3_, PM_10_, and SO_2_) from one measurement station in Reykjavik (GRE).

### Strengths

To our best knowledge, the Reykjavik capital area population is the largest population so far that has been investigated concerning possible adverse health effect of H_2_S exposure where hospital data is used as an outcome. The comprehensive hospital and population registries and the general use of personal identification numbers also strengthen the study, as they allowed us to eliminate readmission and revisits to an acceptable level. The National Roster was used to obtain the population data in each section as well as for the information on location of the patients within the sections attending the hospital, LUH, thus derived from the same source. As the LUH is the only health care institution in the Reykjavik capital area offering acute hospital and emergency department service to the population in the area, there is no competition from other similar health care institutions. However, services are also provided by general practitioners and medical specialists in out-of-hospital offices.

The estimation of the H_2_S exposure in the different sections of the Reykjavik capital area was done by simple model, as the main variables of wind direction and speed, and solar radiation are routinely measured by a governmental institution EAI independently from the continuous accumulation of the outcome information at the LUH. The model estimated H_2_S concentrations in five different sections of the city, which is an improvement from exposure estimates from a single monitoring station in the Reykjavik area, as it gives a more individual-based estimation of exposure. The width of the plume and importance of the distance from the source (the inhabited zones in Reykjavik capital area range 20 to 30 km from source) were taken into account, and have been discussed previously [31].

The methods used in the present study follow the well known, widely accepted, and documented approaches [35].

### Limitations

The exposure data is derived from a simple model for the H_2_S exposure applied in five sections of the Reykjavik capital area, instead of containing data on individual exposure. Also, it is known, that individuals are exposed to air pollution in various other places than their home. This was nonetheless not taken into account whereas information on the patient’s residence was only available data, so some misclassification of H_2_S exposure is possible. Additionally, misclassification of calculated H_2_S exposure is plausible but should be minimal as the difference between the measurements at GRE and the model calculations is small, helped by the fact that nearly zero values occur when the wind direction is not from the east. However, this approach using modelled H_2_S estimations is an advance from the use of concentration measurements obtained from only one measurement station in the Reykjavik capital, as has been used in previous studies [9,16]. Also, we are adjusting for residential distance from main traffic roads as a surrogate for other daily levels of individually measured airborne pollutants, which improved the model fit and gave a better adjustment than measured concentrations from one measurement station (GRE).

In the present study, we were not able to adjust for social variables, or premorbid condition of those with emergency hospital visits. The relative risk sizes found are very small and, even if statistically significant because of the large sample size, they could possibly be accounted for by a small amount of unknown confounding. Detailed investigation of the role of these important factors awaits future studies.

Our study is conducted on material originating from a single academic health care institution in a capital area, which may limit the generalizability of the results; however, the hospital and the ED are together the only institution of their kind serving the catchment population as a community hospital, rendering the study population-based. The characteristics of the population are known and the population is relatively homogenous, being 95-99% white Caucasian [24], and there is a uniform financing of the health-care and insurance.

We tested several approaches in the analyses of the association of the H_2_S exposure and the many components of the outcome measures in an attempt to yield as much knowledge as possible from our data set. The high number of calculations performed may give rise to concern due to multiple comparison problems; however, it has been argued that no adjustments are needed for these [41].

The increased risk for emergency hospital visits with HD as primary diagnosis seemed to be marked for those 73 years of age and older and the population data was restricted to those 18 years and older. This limits the generalizability of the results with regard to age. Another limitation is the relatively small number of cases with diagnoses of respiratory diseases and stroke, rendering analyses of these outcomes statistically underpowered. This is an inherent weakness for studies in small populations. The counting of admissions and visits to LUH were restricted to attendances where HD, respiratory diseases, and stroke were a primary diagnosis of the individuals; thus we were not able to analyse all attendances to LUH. The quality of the routine medical diagnoses at LUH has not been evaluated in a separate study, a weakness that this study shares with most other studies relying on hospital records. Finally, according to the diurnal distribution of the admissions and visits in the study, it was not realistic to achieve a narrower time frame than 24 hours in the association analysis.

## Conclusions

The results from this study indicate an increase in hospital admission and emergency department visits with heart disease as primary diagnosis associated with H_2_S concentrations exceeding 7.00 μg/m^3^ the same day, more pronounced among males and among those 73 years and older. The associations were also seen with delay of two or four days. The same-day associations were gradually increasing through higher percentiles of exposure in a dose-response manner. These results were adjusted for gender, age, season, traffic-related pollution, and number of lags with generally accepted and acknowledged methods. These results are further supported by a previous study in the same setting showing increased general mortality when 24-hour H_2_S concentrations exceed 7.00 μg/m^3^.

## Supporting Information

S1 Fig Daily number of emergency hospital visits with heart diseases, respiratory diseases, and stroke as primary diagnosis in the Reykjavik capital area over the study period 1 January, 2007 to 30 June, 2014. (TIFF)

S2 Fig Daily 24-hour concentrations of H_2_S in μg/m^3^ within modelled sections A to E of the Reykjavik capital area in 2009. The horizontal line indicates the 85 percentile limit of 7.00 μg/m^3^.
(TIFF)

S3 Fig Daily 24-hour concentrations of H_2_S in μg/m^3^ within sections A to E of the Reykjavik capital area in November 2009. The horizontal line indicates the 85 percentile lower limit 7.00 μg/m^3^.
(TIFF)

S4 Fig Hourly number of emergency hospital visits with heart diseases, respiratory diseases, and stroke as primary diagnosis in the Reykjavik capital area during 2009. (TIFF)

S1 Model Calculations Description of hydrogen sulfide concentrations modelling for five different sections of the Reykjavik capital area of Iceland. (PDF)

S1 Table Crude results for associations between daily emergency hospital visits with heart diseases, respiratory diseases and stroke as primary diagnosis and H_2_S concentrations exceeding 7.00 μg/m^3^ for lags 0-4 (adjusted for seasonality only). (DOCX)

S2 Table Associations between daily emergency hospital visits with heart diseases, respiratory diseases, and stroke as primary diagnosis for 7.00 μg/m^3^ changes in H_2_S concentrations (introduced as continuous variable) in fully adjusted models for lags 0-4. (DOCX)
