# Supplementary figures and images for: Association between Daily Hydrogen Sulfide Exposure and Incidence of Emergency Hospital Visits: A Population-Based Study
Source: PLoS One. 2016 May 24;11(5):e0154946. doi: 10.1371/journal.pone.0154946 (PMC4878737; doi:10.1371/journal.pone.0154946)

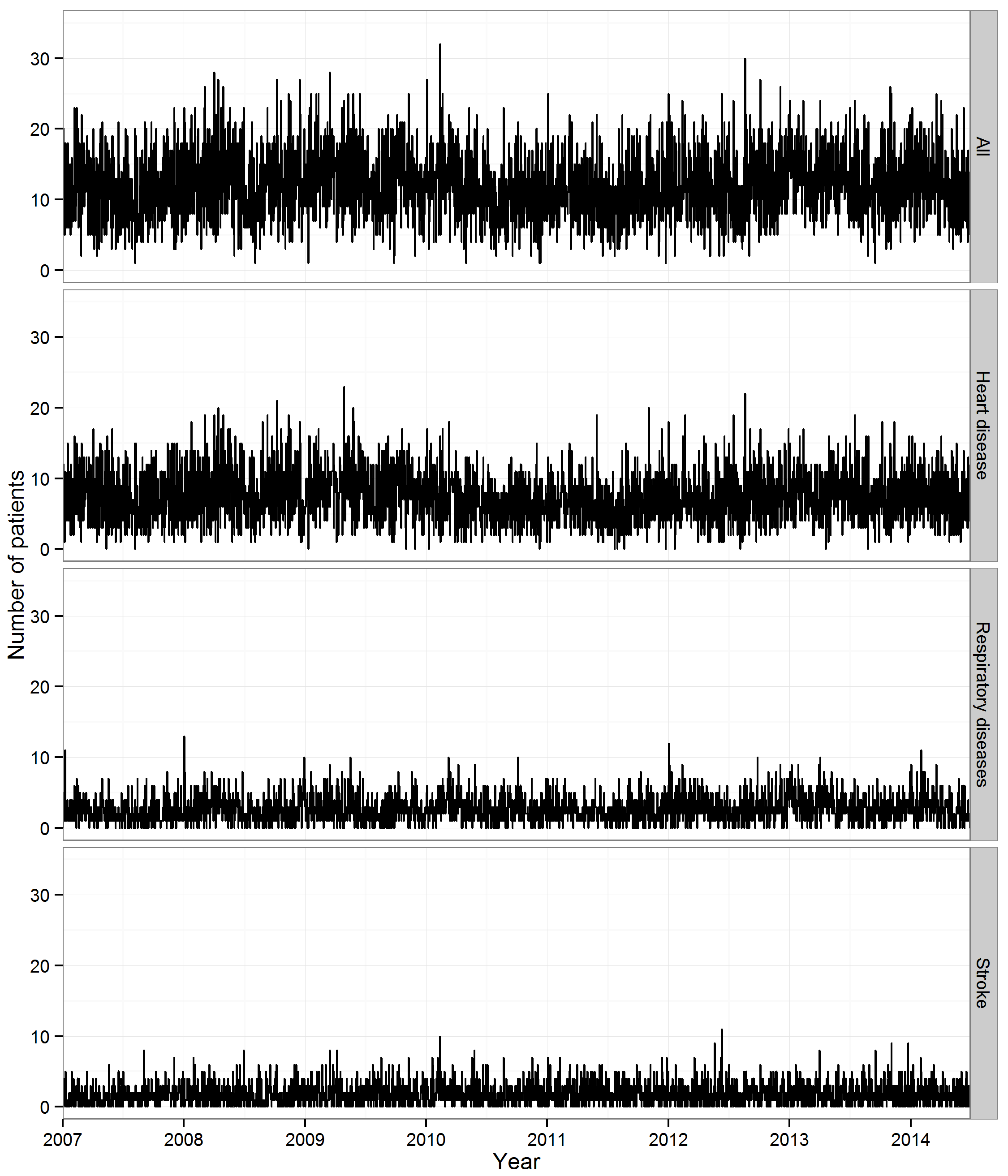

Supplement: S1 Fig — (TIFF) [file pone.0154946.s001.tiff]

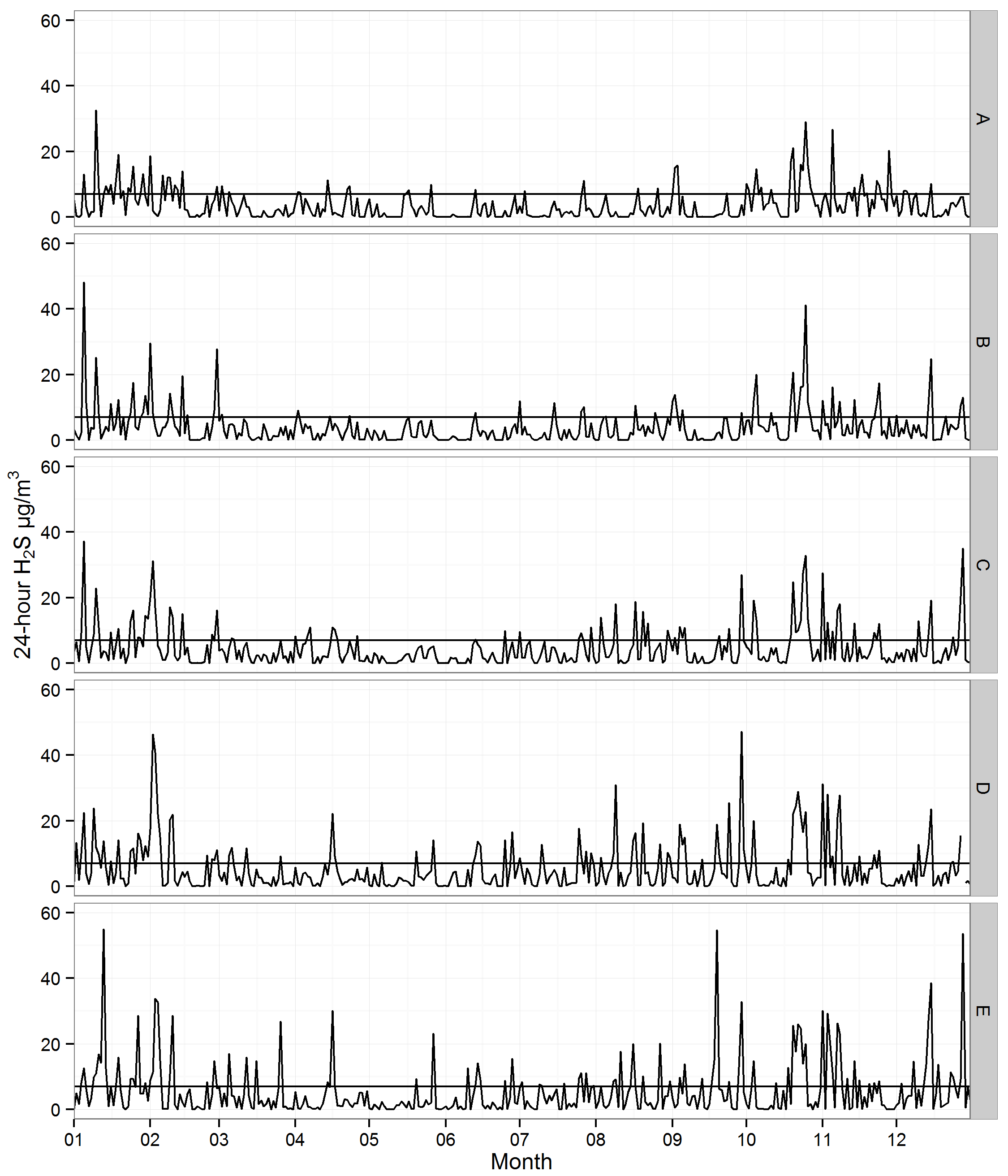

Supplement: S2 Fig — The horizontal line indicates the 85 percentile limit of 7.00 μg/m3. (TIFF) [file pone.0154946.s002.tiff]

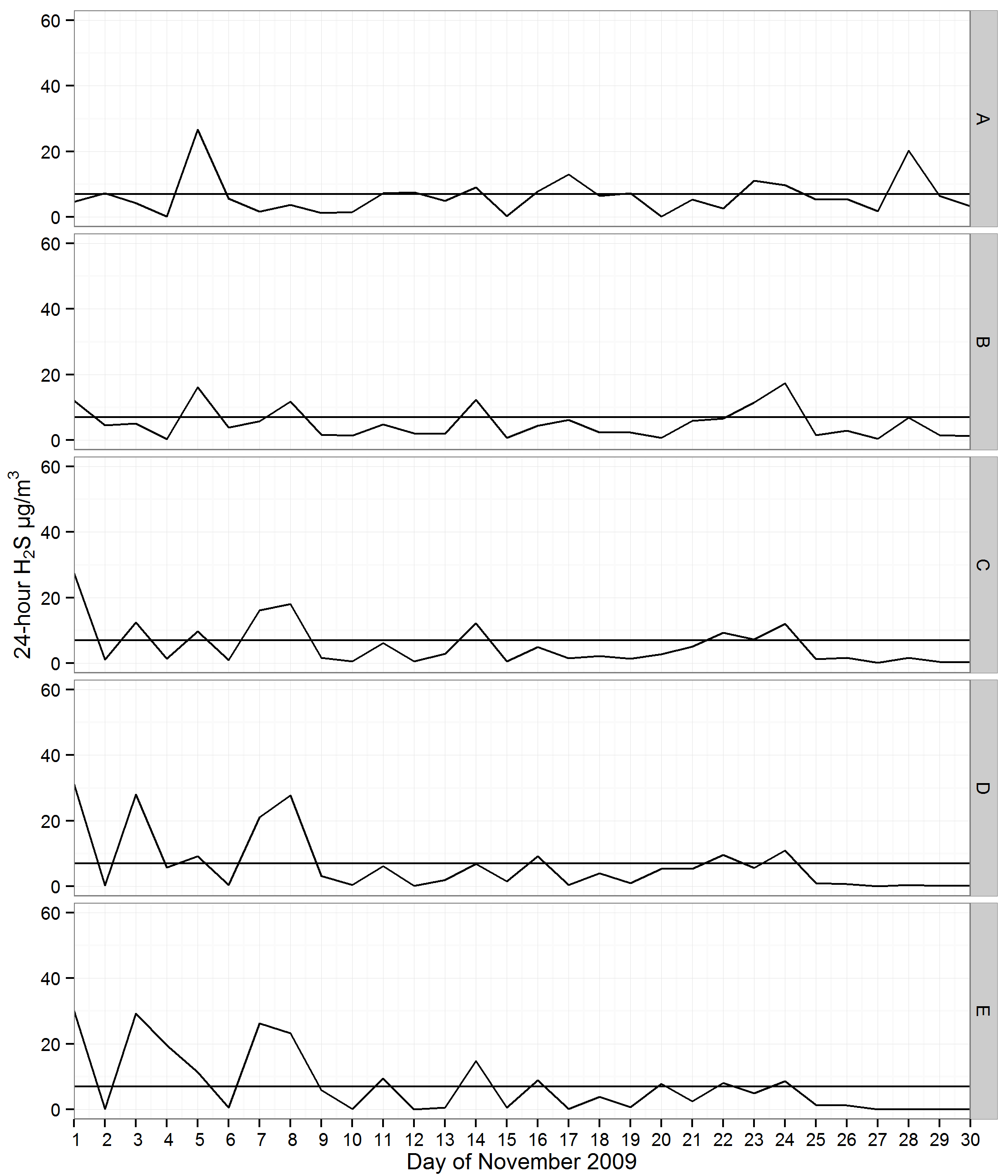

Supplement: S3 Fig — The horizontal line indicates the 85 percentile lower limit 7.00 μg/m3. (TIFF) [file pone.0154946.s003.tiff]

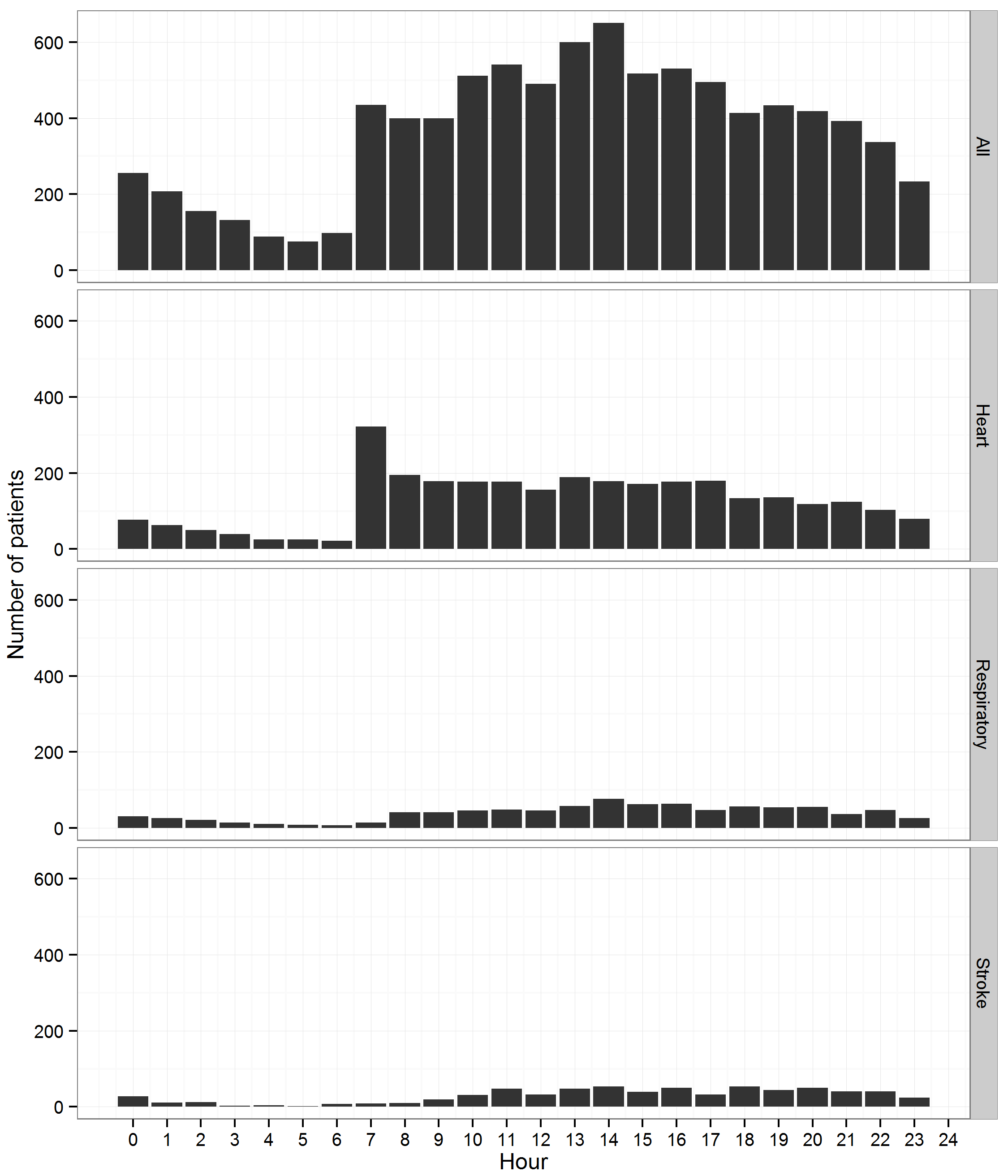

Supplement: S4 Fig — (TIFF) [file pone.0154946.s004.tiff]
