# Supplementary material for: Association between Daily Hydrogen Sulfide Exposure and Incidence of Emergency Hospital Visits: A Population-Based Study
Source: PLoS One. 2016 May 24;11(5):e0154946. doi: 10.1371/journal.pone.0154946 (PMC4878737; doi:10.1371/journal.pone.0154946)
Supplement: S1 Model Calculations — (PDF) [file pone.0154946.s005.pdf]

Modeling H_2S concentration citywide

Throstur Thorsteinsson

Environment and Natural Resources & Institute of Earth Sciences, University of Iceland, Sturlugata 7, 101 Reykjavik, Iceland

Abstract. Measurements of H_2S in the Reykjavik capital area are only available from one station, Grensasvegur station (GRE), for the whole period between January 2003 and July 2014. At another location, the Hvaleyrarholt station (HEH) data are available for the years 2007-2011. To estimate exposure in different parts of the Reykjavik capital area a simple model was used to calculate the concentration of H_2S. The model covered a 50° sector from the Hellisheidi power plant, which includes the densest population areas, in 10° sections. The densest population is between 20 and 30 km from the power plant, and therefore no dependence on distance is included. H_2S pollution is very difficult to predict, but this model, being simple, is comparable to many of the larger modeling work that has been tested for the area.

INTRODUCTION

Exposure to pollutants depends on location and time. H_2S pollution in the Reykjavik capital area, from a geothermal power plant in the vicinity (25 km east of city center), is highly dependent of wind direction and speed (3).

Having only one H_2S measurement station, with a long continuous record within the Reykjavik capital area, the spatial resolution is very low. We therefore developed a method to approximate exposure values within the city based on wind speed and wind direction data.

The concentration of airborne pollution from a point source depends on wind direction, wind speed, and several other factors that determine the air stability. However, for the concentration of H_2S to be high at some distance, here, over 20 km from the source, the atmosphere has to be fairly stable. In that case, the pollution travels as a relatively narrow plume. The well-known inverse dependence on wind speed, $1/u$, where u is wind speed, for the concentration demonstrates the importance of wind speed. That importance is further emphasized by the fact that wind speed is a crucial variable in determining the air stability [1].

METHODS AND DATA

Data from the measurement station at Grensasvegur (GRE) was used in this analysis. The station measures various air pollutants and meteorological variables, including H_2S, wind speed, and direction. Data from Hvaleyrarholt (HEH) station is available for 2007–2011 and the

concentration there is modeled as well. Only a short period of time, using GRE data, is used to constrain the model.

The direction, θ, from the power plant to the Reykjavik capital area covers approximately the range between 90° (aluminum smelter in Straumsvik) to 140° (Leirvogur in Mosfellsbae) (Figure A).

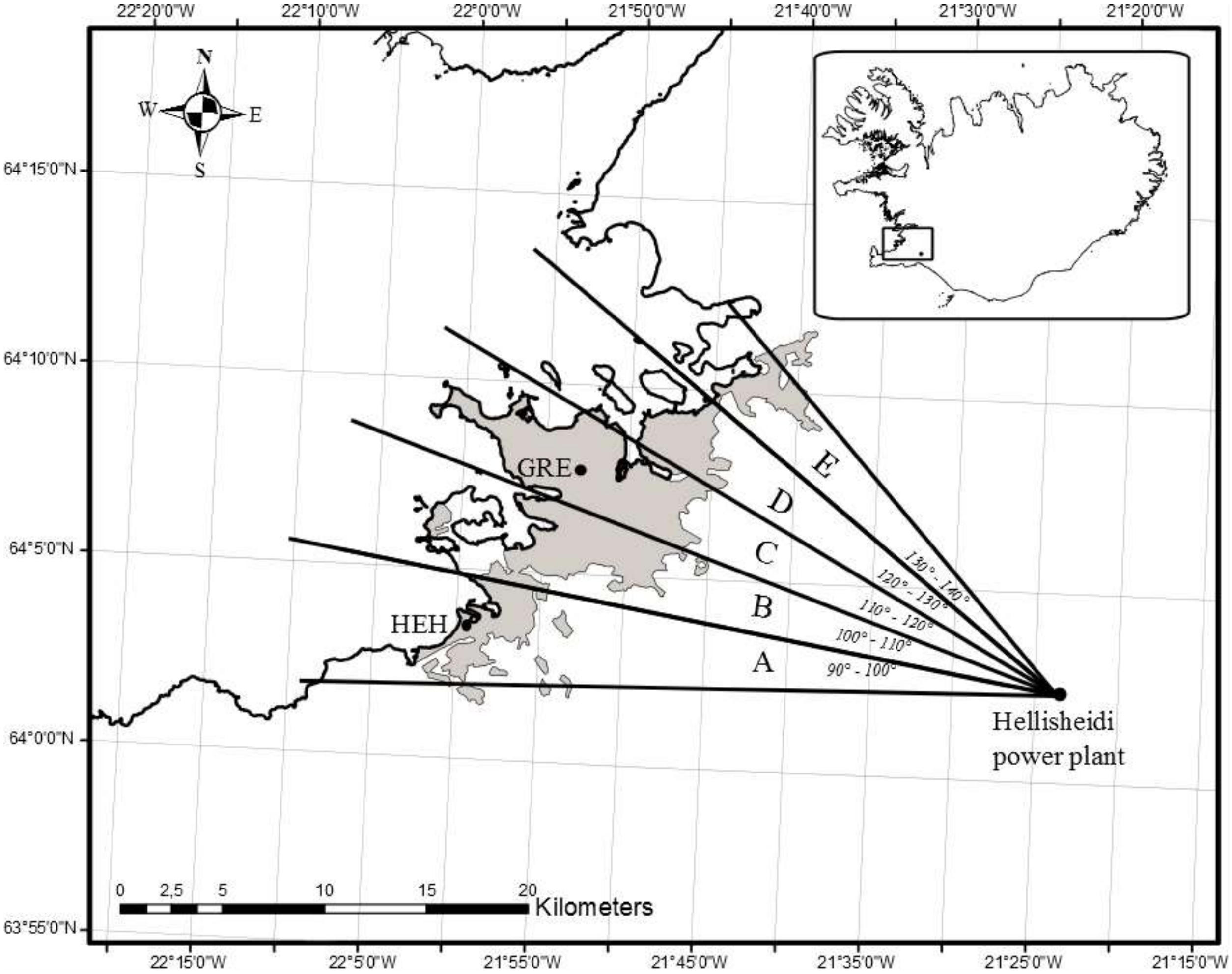

Figure A. Map showing the area covered for θ between 90° and 140°. Also shown is the location of the geothermal power plant at Hellisheidi, and the measurement stations at Grensasvegur (GRE) and Hvaleyrarholt (HEH).

CALCULATIONS

The concentration at a given distance (here between 20 and 30 km away from the source), and direction θ is given as,

$$C = \frac{100}{u} \, exp\left(-\frac{|\theta-\phi|}{\Phi}\right) exp\left(-\frac{R}{R_0}\right) + C_t,$$

where u is wind speed, θ is direction of calculation point, φ is the wind direction, Φ is the angular width, R is incoming radiation and R_0 is the scaling factor for the effect of solar radiation. Here R_0= 35 W/m^2 (Figure B), and Φ=7° (Figure C).

For the time resolution, 30 minutes, the residual pollution at each time step is calculated as

$$C_t = C_{t-1} exp(-u),$$

where u is wind speed (here considered normalized with 1 m/s).

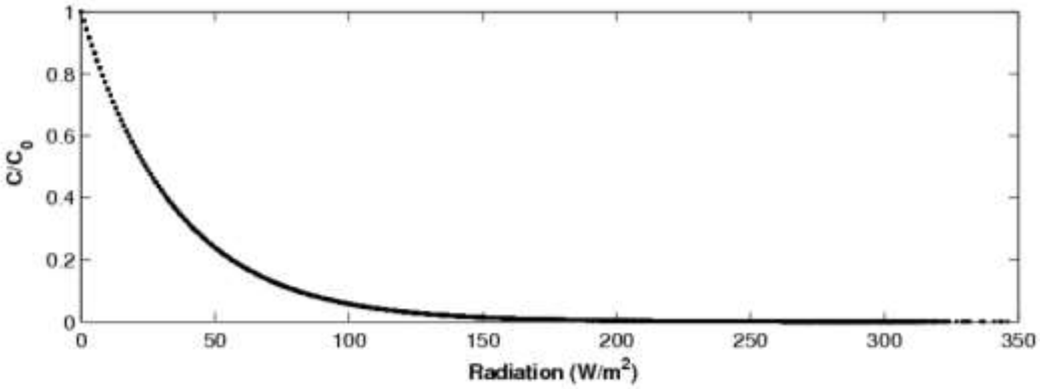

Figure B. The effect of solar radiation on the concentration.

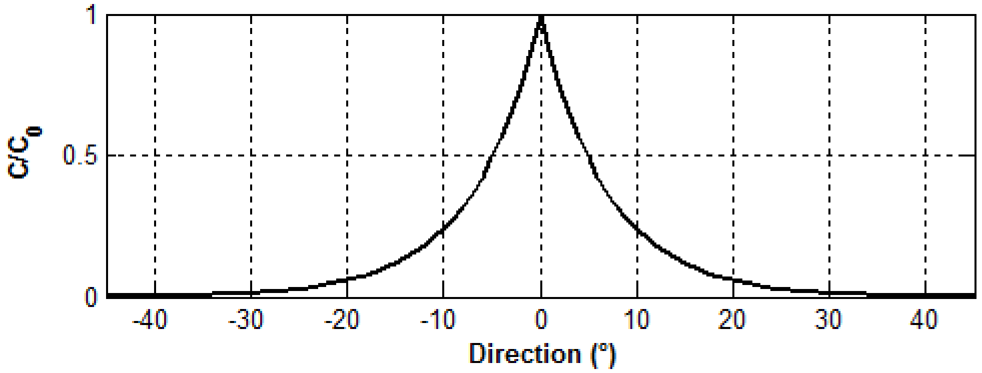

Figure C. The decay of the plume concentration away from the plume center, ϕ.

The width of the plume is determined from calculations using a Gaussian plume, Pasquill-Gifford model (1, 2), at 25 km from the source under stable conditions (3). Stable is the most common conditions when the H_2S is above 50 µg/m^3 with over 60% of the cases in 2007-2009, followed by inversion, which has an even narrower width, with over 30% of the cases (3). The solar radiation function is applied since when H_2S exceeds 50 µg/m^3 it is after sunset in more than 75% of the cases (3).

RESULTS

Calculations were done for the Reykjavik capital area (Figure A). The model results are compared with measurements from GRE and HEH stations using the daily average concentration values as used for the admission data (Figures D and E); model calculated for every hour. The difference between the measurements and the model is minimum, helped by the fact that nearly zero values occur when the wind direction is not from the east. Therefore we examine the low and high values separately. The model tends to under predict the high concentration values (Figs. 4 and 5; bottom right), which can be explained by the model taking into account average conditions, not the conditions that lead to peak concentration. There is less deviation for the lower values (Figs. 4 and 5; bottom left).

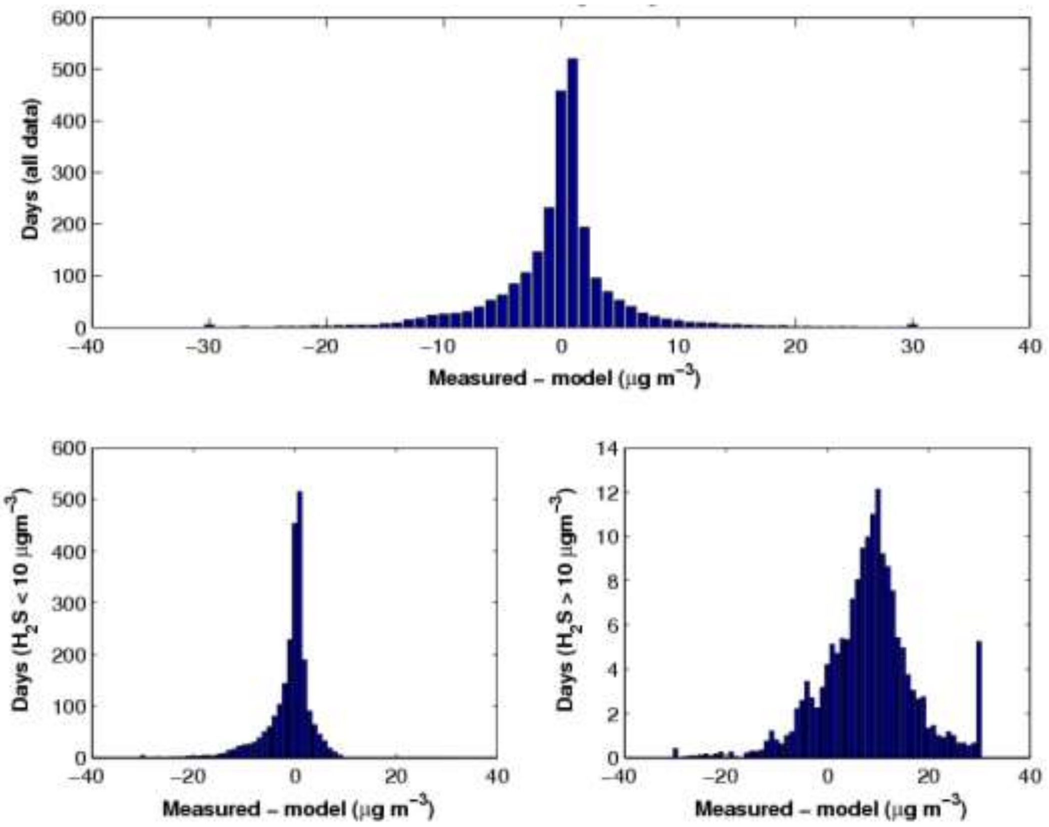

Figure D. Difference between measured and modelled H_2S concentration at GRE station using 24-hour average values. The difference for all values (top), and for measured values smaller (bottom left) and larger 10 µg/m^3 (bottom right) than are shown.

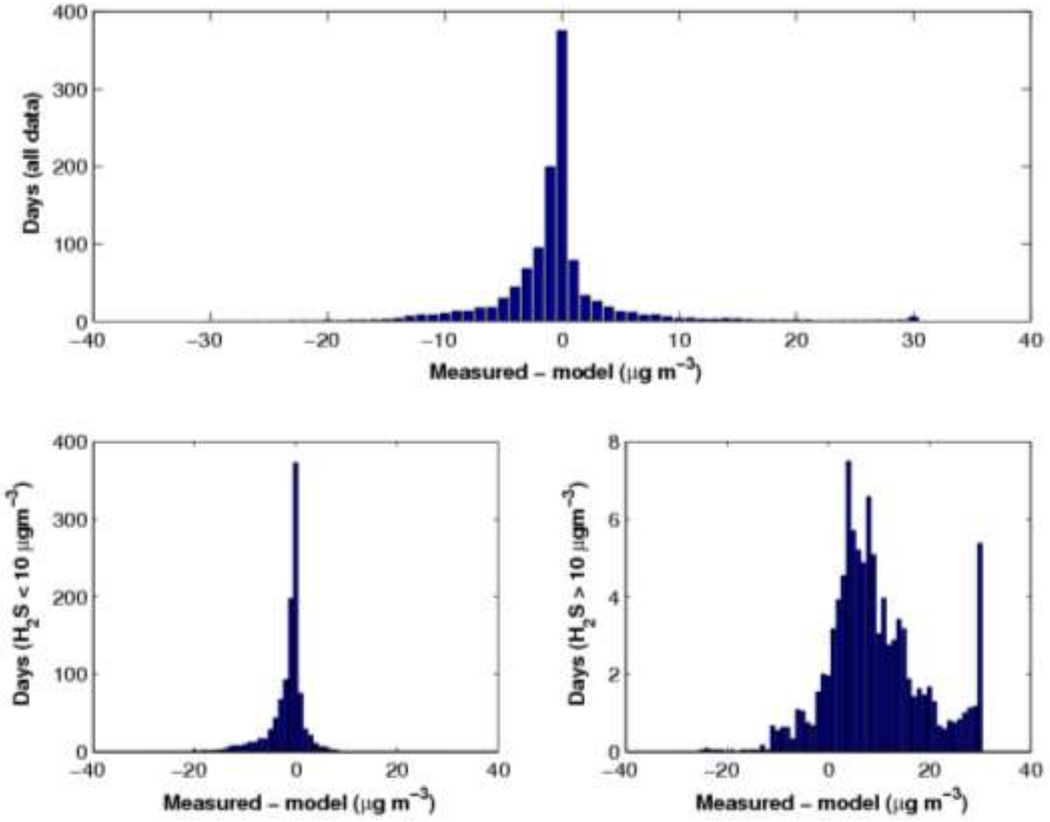

Figure E. Difference between measured and modelled H_2S concentration at HEH station using 24-hour average values. The difference for all values (top), and for measured values smaller (bottom left) and larger than 10 µg/m^3 (bottom right) are shown.

DISCUSSION

We know that wind direction and speed are the dominant variables when it comes to H_2S concentration in the Reykjavik capital area (3). The concentration also depends on the distance from the source, which is not included in these calculations. There are at least couple of reasons for not including this effect. First, most of the population is concentrated on a semi-circle between 20 and 30 km from the Hellisheidi power plant. Secondly, spot measurements in the area have shown that the concentration is often quite variable, independent of distance (within the range of 20-30 km from the power plant), and that topography seems to have an influence; although that has not been quantified.

Reference list

1. Gifford F, Hanna S. Modelling urban air pollution. Atmospheric Environment (1967). 1973;7(1):131-6.
2. Kielland G. Guidance report on preliminary assessment under EC air quality directives", 1998. European Environment Agency, 11.
3. Thorsteinsson T, Hackenbruch J, Sveinbjörnsson E, Jóhannsson T. Statistical assessment and modeling of the effects of weather conditions on H2S plume dispersal from Icelandic geothermal power plants. Geothermics. 2013;45:31-40.
