# Supplementary material for: Association between Daily Hydrogen Sulfide Exposure and Incidence of Emergency Hospital Visits: A Population-Based Study
Source: PLoS One. 2016 May 24;11(5):e0154946. doi: 10.1371/journal.pone.0154946 (PMC4878737; doi:10.1371/journal.pone.0154946)
Supplement: S1 Table — (DOCX) [file pone.0154946.s006.docx]

**S1 Table.** Crude results for associations between daily emergency hospital visits with heart diseases, respiratory diseases and stroke as primary diagnosis and H_2_S concentrations exceeding 7.00 µg/m^3^ for lags 0-4 (adjusted for seasonality only).

| **Lag** | **RR** | **95% CI** | **Diagnosis** |
| --- | --- | --- | --- |
| 0 | 1.123 | 0.965, 1.306 | Heart disease |
| 1 | 1.117 | 0.960, 1.300 | Heart disease |
| 2 | 1.126 | 0.969, 1.310 | Heart disease |
| 3 | 1.138 | 0.978, 1.323 | Heart disease |
| 4 | 1.115 | 0.958, 1.297 | Heart disease |
| 0 | 1.082 | 0.848, 1.380 | Respiratory disease |
| 1 | 1.114 | 0.875, 1.418 | Respiratory disease |
| 2 | 1.090 | 0.855, 1.388 | Respiratory disease |
| 3 | 1.112 | 0.873, 1.416 | Respiratory disease |
| 4 | 1.136 | 0.893, 1.444 | Respiratory disease |
| 0 | 1.123 | 0.841, 1.500 | Stroke |
| 1 | 1.072 | 0.799, 1.437 | Stroke |
| 2 | 1.146 | 0.757, 1.526 | Stroke |
| 3 | 1.122 | 0.715, 1.497 | Stroke |
| 4 | 1.053 | 0.785, 1.414 | Stroke |
