# Supplementary material for: Association between Daily Hydrogen Sulfide Exposure and Incidence of Emergency Hospital Visits: A Population-Based Study
Source: PLoS One. 2016 May 24;11(5):e0154946. doi: 10.1371/journal.pone.0154946 (PMC4878737; doi:10.1371/journal.pone.0154946)
Supplement: S2 Table — (DOCX) [file pone.0154946.s007.docx]

**S2 Table.** Associations between daily emergency hospital visits with heart diseases, respiratory diseases, and stroke as primary diagnosis for 7.00 µg/m^3^ changes in H_2_S concentrations (introduced as continuous variable) in fully adjusted models for lags 0-4.

| **Lag** | **RR** | **95% CI** | **Diagnosis** |
| --- | --- | --- | --- |
| 0 | 1.087 | 0.874, 1.343 | Heart disease |
| 1 | 0.893 | 0.714, 1.118 | Heart disease |
| 2 | 1.149 | 0.932, 1.426 | Heart disease |
| 3 | 0.932 | 0.751, 1.157 | Heart disease |
| 4 | 1.000 | 0.808, 1.230 | Heart disease |
| 0 | 0.791 | 0.537, 1.173 | Respiratory disease |
| 1 | 0.768 | 0.509, 1.157 | Respiratory disease |
| 2 | 0.808 | 0.541, 1.213 | Respiratory disease |
| 3 | 0.796 | 0.533, 1.189 | Respiratory disease |
| 4 | 1.213 | 0.838, 1.759 | Respiratory disease |
| 0 | 1.118 | 0.709, 1.759 | Stroke |
| 1 | 0.919 | 0.566, 1.484 | Stroke |
| 2 | 1.840 | 1.181, 2.878 | Stroke |
| 3 | 0.925 | 0.575, 1.494 | Stroke |
| 4 | 0.658 | 0.409, 1.065 | Stroke |
